# Fatigue Behaviour and Its Effect on the Residual Strength of Long-Fibre-Reinforced Thermoplastic PP LGF30

**DOI:** 10.3390/ma16186174

**Published:** 2023-09-12

**Authors:** Christian Witzgall, Marc Gadinger, Sandro Wartzack

**Affiliations:** Engineering Design, Friedrich-Alexander-Universität Erlangen-Nürnberg, 91058 Erlangen, Germany; gadinger@mfk.fau.de (M.G.); wartzack@mfk.fau.de (S.W.)

**Keywords:** fatigue, residual strength, long-fibre-reinforced plastics, PP LGF30, material data

## Abstract

It is undeniable that mechanical properties, such as the stiffness or residual strength of fibre-reinforced thermoplastics, are adversely affected by fatigue damage caused by cyclic loading. In order to quantify and predict this damage influence, a calculation approach was developed in the past for the subgroup of short-fibre-reinforced thermoplastics. In order to test and expand the applicability of this approach to the field of long-fibre-reinforced thermoplastics, the decrease in mechanical properties is investigated experimentally in this paper using PP LGF30, propylene reinforced with long glass fibres, as an example. The paper describes both the fatigue behaviour and the residual strength of the material after fatigue damage. A decrease in the residual strength of up to about 35% could be recorded. The paper also presents a modelling approach that predicts the orientation-dependent fatigue strength of the material, and furthermore allows for the calculation of its residual strength as a function of fatigue damage. The novelty of the contribution lies in the continuous modelling of fatigue behaviour for arbitrary oriented samples of long-fibre-reinforced thermoplastics and also in the prediction of its residual strength depending on previously induced fatigue damage.

## 1. Introduction

Discontinuous fibre-reinforced plastics have become indispensable in modern vehicle design. As known composite materials, they have excellent lightweight properties such as good stiffness at a low density. High energy absorption also makes them interesting for applications with crash relevance. Discontinuous fibre-reinforced thermoplastics are also used in consumer applications such as housings for power tools or similar applications. 

**Figure 1:**
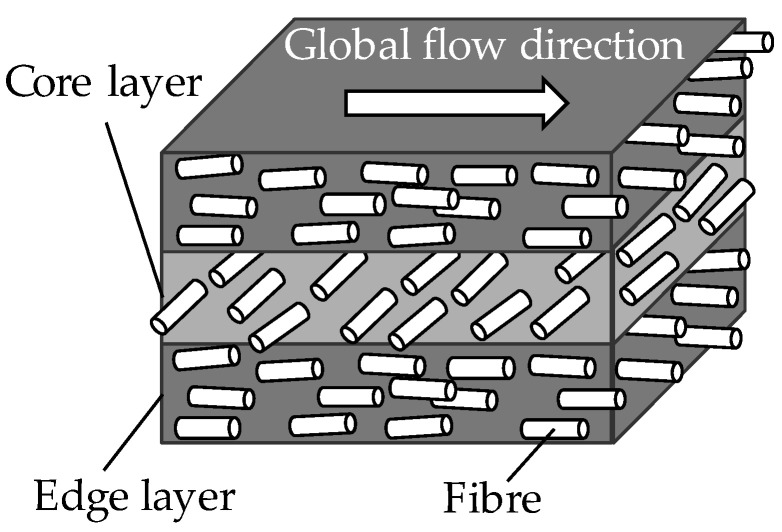
Layer formation of fibre orientation in injection moulded discontinuous fibre-reinforced thermoplastics.

The thermoplastic FRP parts are most often produced by injection moulding, making them attractive because of the ease of fabrication and relatively low cost [1]. The process conditions during injection moulding have a great influence on the fibre orientation. Therefore, a simulation of the manufacturing process is carried out before the actual structural simulation [2]. The knowledge gained about the fibre orientation is used in the structural simulation to correctly map effects such as anisotropy. In injection-moulded sheet composites, the distribution of fibre orientation is typically pronounced in three characteristic layers (edge layer, core layer, edge layer). Filling speed, processing conditions, material behaviour as well as concentration and geometry of the fibres influence this distribution differently. Figure 1 schematically shows the formation of the layers in the three-layer model, which is often used in a simplifying way to model the layer formation. In fact, there are other models, some of which make finer divisions and take into account up to six, sometimes seven layers [3]. However, their knowledge gain is rather small in relation to the additional effort.

Due to the flow-dependent velocity profile, different fibre orientations occur in plate-shaped components made of discontinuous fibre-reinforced thermoplastics. The melt is injected into the cavity from the sprue. Due to the swelling effect in the sprue, the plastic mass spreads parabolically with a closed flow front until the entire width of the cavity is filled by the resulting expansion flow [4,5]. 

The expansion flow leads to a preferential orientation of the fibres in the core layer of the component. The fibres orient themselves transversely to the flow direction and experience only a very low shear rate, which has no influence on their orientation. Pavsek also states that the low velocity gradient favours the orientation in the tool centre [4] and strongly increases the fibre density in the core layer [6].

**Figure 2:**
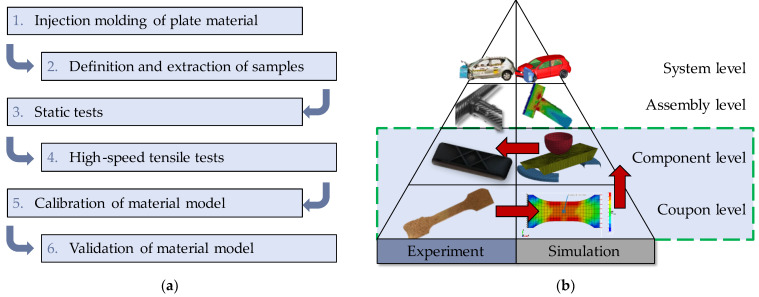
Process of material data determination for fibre-reinforced thermoplastics (**a**) and Rouchon’s pyramid of testing (**b**).

Even when utilising an injection moulding simulation, high-quality structural simulation results can only be determined if precise material data are available. On the one hand, this requires high-quality test and measurement methods, but also suitable sample material. Characteristic values determined for specimen material that is as good as new and that is only produced for material testing do not provide any information about the performance of the material after it has survived a certain period of use. This procedure, which is also shown in Figure 2, is currently common practice. Plates are injection-moulded from granules. Specimens are defined in these plates and often taken by milling or waterjet cutting. Different orientations of the specimens with respect to the main flow direction are produced. With the specimens obtained in this way, static tests are first carried out, which reveal a basic knowledge of the material behaviour. Subsequently, high-speed tensile tests are performed to characterise the strain-rate-dependent material behaviour. A material model is calibrated with these material data. All these experiments happen at the coupon level in Rouchon’s testing pyramid, since coupon testing is simple, inexpensive, and feasible in large numbers [7]. For the final validation of the material model, the test moves to a higher test level, the component level. 

Components in the field, however, are subjected to operational loads during their service life, which leads to material fatigue before, in the case of a vehicle, destructive crash loading occurs. This material deterioration leads to a decrease in stiffness and residual strength [8], which requires special attention when calculating these particular loading conditions, even if high stress amounts are not expected, such as with Hertzian pressures of several 1000 MPa [9]. For the subgroup of short-glass-fibre-reinforced thermoplastics, an approach has been developed and published that allows for continuous modelling of the fatigue behaviour over the orientation direction and consideration of the fatigue damage in the crash simulation. 

However, it is necessary and advantageous to also include the group of long-fibre-reinforced thermoplastics in the consideration as the use of longer fibres leads to a higher reinforcing effect in the material, resulting in better mechanical properties [10]. Furthermore, long-fibre-reinforced thermoplastics are used in several different applications and branches such as hatchbacks, door panels or bumper beams and front end modules in automotive engineering [11], or different types of casings in consumer electronics, power tools and industrial equipment.

This paper therefore attempts to generalise this approach and apply it to the material class of long-fibre-reinforced thermoplastics. As the combination of polypropylene as matrix material is often found due to its beneficial processing properties [12] with long glass fibres as reinforcement, PP LGF is the most studied long-fibre-reinforced thermoplastic [10], mostly driven by its wide use in the automotive industry. 

The paper addresses the scientific question: Is the approach developed for short-fibre-reinforced thermoplastics also able to predict the residual strength of long-fibre-reinforced thermoplastics? The novelty of the contribution lies in the continuous modelling of fatigue behaviour for arbitrary oriented samples of long-fibre-reinforced thermoplastics and furthermore in the prediction of its residual strength depending on previously induced fatigue damage.

## 2. Modelling Residual Properties after Fatigue Damage

**Figure 3:**
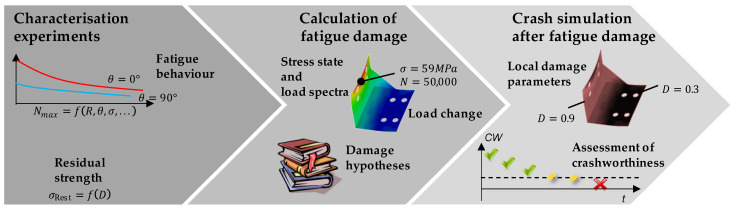
Steps to determine residual strength after fatigue damage in short-fibre-reinforced thermoplastics.

In previous work, an approach was developed to investigate the fatigue behaviour of short-fibre-reinforced thermoplastics and the influence of material fatigue on the residual strength [13,14,15]. The overall approach aims at predicting the crash-relevant material behaviour and therefore also takes into account the effects of the strain rate on the failure stress. Its main steps are shown in Figure 3 and described further within the following sections. 

### 2.1. Determination of Fatigue Damage

The increase in damage is obtained from the in situ measurement of stiffness using digital image correlation during the cyclic tests. The material experiences a non-linearly increasing damage [16]. The damage is denoted by the parameter *D* and corresponds to the loss of stiffness Δ*S* compared to its initial value [17]. It is defined as:

(1) $$D=\Delta S(n)=\frac{E\left(n\right)}{E_{0}}={\left(1-{\left(\frac{n}{N_{max}}\right)}^{A}\right)}^{B}$$

where *E* is the current respective initial modulus of elasticity, *n* is the current number of oscillations and *N*_max_ is the maximum load-bearing capacity. *A* and *B* are model parameters to be calibrated, which describe the increase in damage over the service life. 

### 2.2. Modelling of Residual Strength

In the failure modelling of SFRT materials, the Tsai–Hill criterion is a widely used failure criterion. The stress tensor components are combined into an interactive failure criterion that, when a threshold level is reached, signifies failure. Due to their simplicity of use, interactive criteria are frequently used in Finite Element simulations to predict the failure of composite materials, even though they do not provide information on failure mechanisms [18]. Tsai–Wu and Puck are two other relevant criteria; the latter is typically used for laminated composites. The Tsai–Hill criterion, which is developed from Hill’s yield criterion [19,20], is as follows for materials that are transversely isotropic:

(2) $$\frac{\sigma_{∥}^{2}}{\sigma_{∥,max}^{2}}+\frac{\sigma_{⊥}^{2}}{\sigma_{⊥,max}^{2}}-\frac{\sigma_{∥}\cdot \sigma_{⊥}}{\sigma_{∥,max}^{2}}+\frac{\tau_{∥⊥}^{2}}{\tau_{∥⊥,max}^{2}}=1$$

On injection-moulded coupons, the values of the constants $\sigma_{∥,max}$, $\sigma_{⊥,max}$ and $\tau_{∥⊥,max}$ will be ascertained experimentally. The material’s strength is assessed under tensile stress, and the testing directions are relative to the flow direction. The symbols for the flow direction strength, the transverse to the flow direction strength and the shear strength are $\sigma_{∥,max}$, $\sigma_{⊥,max}$ and $\tau_{∥⊥,max}$, respectively.

In order to consider the effect of the damage parameter *D* on the residual strength, an approach according to Paepegem and Degrieck [21,22,23] is used, which was developed for fibre-reinforced plastics in general. This specifies:

(3) $$\sigma_{RT}=X_{T}\cdot {\left(1-D\right)}^{p}$$

The residual tensile strength is denoted as $\sigma_{RT}$, while the tensile stress is denoted as *X_T_*. The calculation is analogous for all parameters of the Tsai–Hill criterion. Based on the investigations of Shokrieh and Lessard in [24], it is determined that the development of the remaining stiffness and strength proceeds with comparable slopes. Therefore, a value below one is recommended for the exponent *p*—otherwise it would be implied that the strength decreases faster than the stiffness. Due to the general nature of this approach, the model could easily be applied to the situation of single-axis tensile tests of short-fibre-reinforced thermoplastics. There is also reason to believe that the model is also suitable for use with long-fibre-reinforced thermoplastics.

## 3. Materials and Specimen Geometry

**Figure 4:**
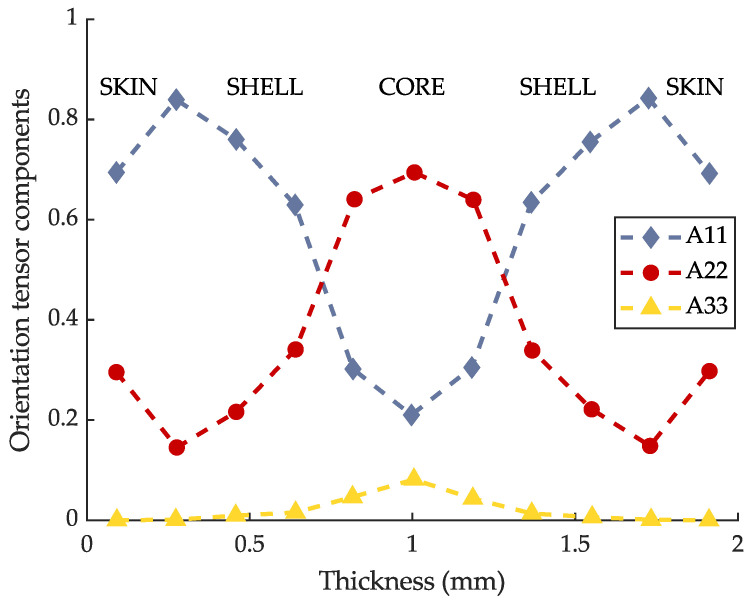
Experimental fibre orientation distribution by micro-CT, according to [25].

The material used for this work is PP LGF30, a polypropylene reinforced with 30% long glass fibres by weight. Long-glass-fibre-reinforced thermoplastics are a subgroup of discontinuous fibre-reinforced thermoplastics whose initial fibre length is 8 to 25 mm. During the manufacturing process, the fibre length is reduced to up to 3 to 5 mm, among other things by breaking the fibres in the extruder screw. However, this is still a significantly greater length than for short fibres, which are only <<1 mm long. The fact that the length of the reinforcing fibres is greater than the usual thickness of thin-walled injection moulded parts (1.5 to 2 mm) results in a particularly layered distribution of the fibres. Orientation in the thickness direction is practically impossible. This is illustrated in Figure 4, which shows the typical fibre orientation over the thickness using the components of the fibre orientation tensor [25]. It can be seen that high values of *a*_11_ occur in areas close to the edge. This means that the fibres are mainly oriented along the main flow direction. In the centre of the component, the value of *a*_22_ dominates, which means that the fibres are mainly oriented transverse to the flow direction. The entries *a*_33_, which indicate an orientation in the thickness direction, play practically no role in long-fibre-reinforced thermoplastics. This leads to an even stronger pronouncement of the core layer than is known for short-fibre-reinforced thermoplastics.

**Figure 5:**
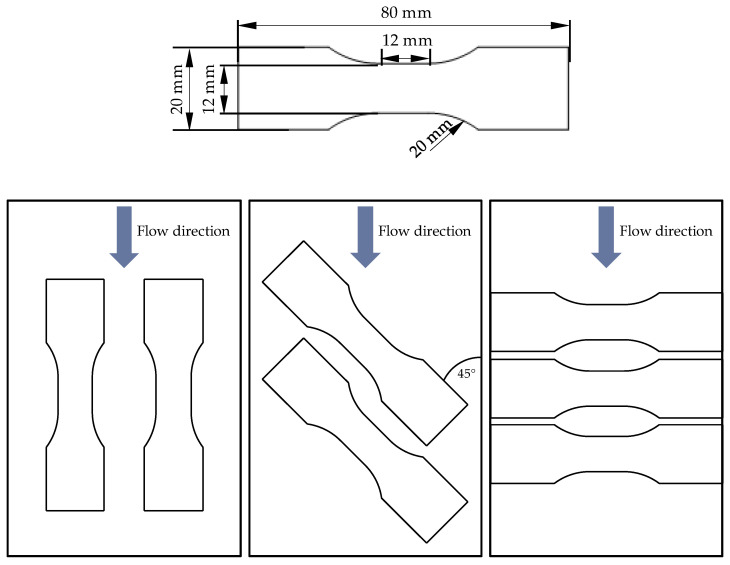
Becker specimen geometry and positioning within plates.

In experimental testing, Becker tension rods according to [26] are used as test specimens, which are taken from 2-mm thick injection-moulded plates of 120 mm × 80 mm by milling (Figure 5). The Becker tension rods were developed specifically for use in high-speed tensile tests and, due to their short parallel area, allow a high strain rate to be achieved even at moderate test speeds. Furthermore, they have a sufficient width in their middle area, which makes an optical evaluation of the strains easily possible [26].

While the use of such short specimens is unusual in the field of cyclic material testing, the use of these specimens allows for future comparison in highly dynamic experiments.

## 4. Experimental Methods

The aim of the experimental investigation is to determine the material behaviour of PP LGF30 in the virgin state, the fatigue behaviour as well as the remaining material characteristics after previous cyclic loading. Accordingly, the experimental test programme is comparable to that previously published on the short-glass-fibre-reinforced PBT GF30:

- Static tensile tests on new specimens to determine the elastic moduli and strengths of the new material with different fibre orientations. All orientations will be repeated 3 times each.
- Destructive cyclic tests to determine the damage progression and the orientation angle-dependent S–N curves, which represent the fatigue behaviour. Overall, 36 specimens are used.
- Non-destructive cyclic tests on individual specimens prior to damage.
- Static tensile tests on pre-damaged specimens for comparison with undamaged specimens and validation of models for predicting residual strength, with overall 13 specimens.

All tests were carried out on the Zwick HCT 25 (Zwick, Ulm, Germany) servo-hydraulic pulser, on which the cyclic, but also the static, material behaviour of the specimens can be investigated. All experiments were carried out at a room temperature of 23 °C. The force and piston travel are measured with an integrated piezoelectric load cell and an inductive displacement transducer. A test speed of 10 mm/min was set for the static tests. The cyclic experiments are carried out with a test frequency of 4 Hz, as has already proven successful for other thermoplastic materials [27]. At this comparably low test frequency, there is not yet a critical temperature increase in the sample, which would be caused by internal friction and hysteresis. As a safeguard, the temperature on the sample surface is nevertheless measured and recorded with an infrared thermometer. This is because from an unwanted temperature increase of 10 °C or more, loss of strength and premature failure of the samples must be expected. The cyclic tests are carried out with a stress ratio *R* of 0.1, as suggested in [28]. The experiments are carried out in a load-controlled manner. 

Both the static and the cyclic tests are evaluated by means of digital image correlation (DIC) [29]. On the one hand, this allows precise stress–strain curves to be generated. On the other hand, an evaluation of the stiffness over the duration of the cyclic experiment is possible in situ. The DIC system used is GOM ARAMIS 3D 4M. While the use of this optical measuring technique is quite trivial in the static experiment, its use and control during the cyclic experiments will be explained in more detail.

**Figure 6:**
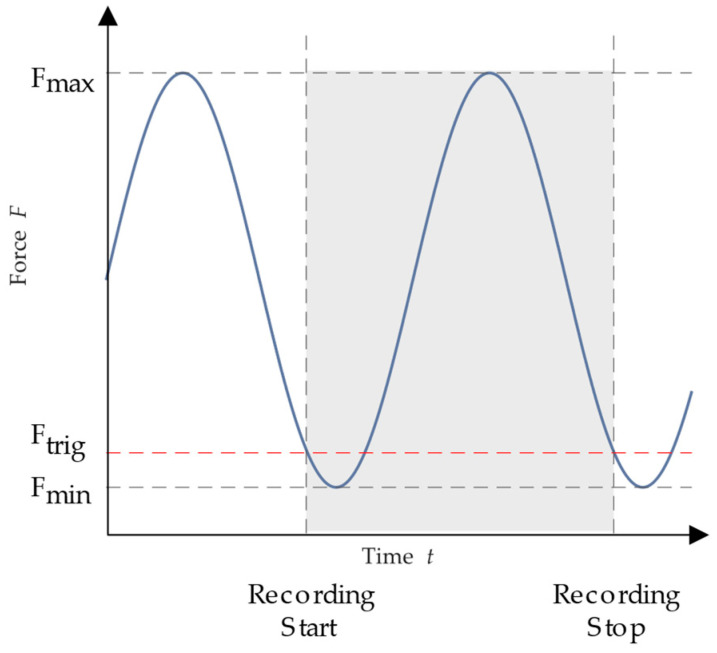
Trigger and pause mode for optical measurement in fatigue experiment.

The long experiment times make it impossible to permanently record the sample’s deformation since the ensuing data volumes would be insurmountably large. Instead, depending on the anticipated total running time, a full loading and unloading of the specimen needs to be recorded at specific intervals. It is possible to start recording based on the observed force by connecting the camera system to the test stand’s force measurement system. Figure 6 depicts a portion of the measurement process. 

A trigger force *F*_trig_ is defined, which should be just above the lower force *F*_u_. A continuous recording with a sampling rate of 120 Hz starts when this trigger force is crossed in a descending direction, that is, with a negative gradient. Until the trigger force is again crossed with a negative gradient, the recording is kept going. There is then a wait of *n* seconds before the next recording may begin. Thus, it is possible to record the whole loading and unloading of the specimen without having to record each cycle. By accurately determining the measurement pause time based on the anticipated overall experiment duration, excessive data are prevented. Based on this, the rise in the stress–strain curve is used by DIN ISO 527-1 [30] to calculate the modulus of elasticity of the nth cycle. In order to achieve the relative decline in stiffness, this is set in reference to the beginning value, which is the modulus of elasticity of the first oscillation.

**Figure 7:**
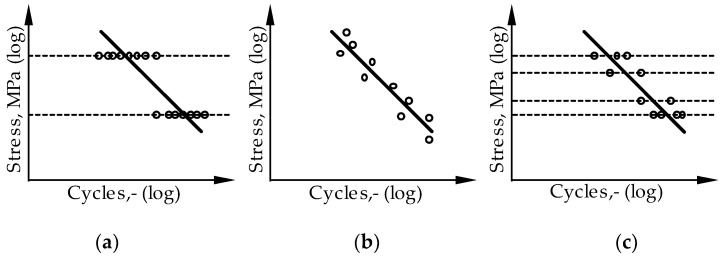
Determination of S–N curves according to [31]: (**a**) horizon method, (**b**) string-of-pearls method, (**c**) mixed mode.

DIN 50100 [31] suggests two different possibilities for determining S-–N curves with the string-of-pearls method and the horizon method. The horizon method means that several tests are carried out on selected stress horizons, Figure 7a [32]. These stress horizons should be as close as possible to the transition areas for short-term or fatigue strength. Figure 7b shows the determination of the S–N curves using the string-of-pearls method, whereby the individual tests are carried out at many different stress horizons and are lined up along the fatigue strength line as if on a string of pearls. When carrying out the string-of-pearls method, a test is started on any load horizon for which a result in the time strength range is assumed. Depending on the test result, subsequent tests are carried out with higher or lower loads in order to investigate the range step by step. Accordingly, an inaccuracy of the first assumption has no influence on the test result. This justifies the special suitability of the bead string method for characterisation tests of materials with only a little available experience. 

In order to combine the advantages of both methods, according to Martin et al. mixed forms are also encountered, in which single or several vibration tests are carried out on more than two stress levels, as can be seen in Figure 7c [32]. This procedure is also used to carry out the tests in this paper.

## 5. Results

### 5.1. Results of Static Testing

**Figure 8:**
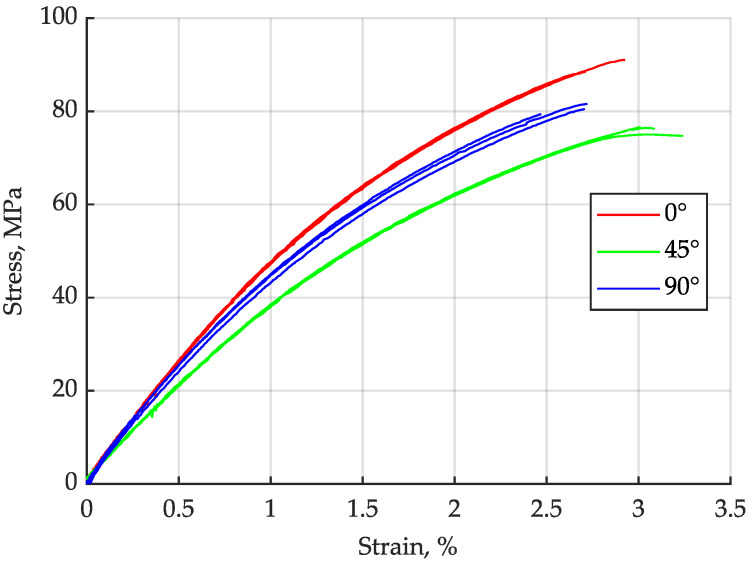
Stress–strain behaviour of PP LGF30.

The results of the static characterisation are shown in Figure 8. As expected, the samples in which the test load acts along the main flow direction of the melt, 0°, show the strongest material behaviour: with a Young’s modulus of 5124 MPa and a strength of 89.2 MPa, the highest strength values are found here. However, the specimens tested transverse to their gating direction are unexpectedly the second strongest orientation. With an even slightly higher average stiffnesses of 5352 MPa and strengths of 80.5 MPa, they are only slightly behind the longitudinal specimens. The lowest stiffnesses or strengths were identified as 4159 MPa and 76.0 MPa for the specimens taken diagonally, 45°, from the plates.

This observed material behaviour is very reminiscent of the properties also found in multi-layer laminates with 0° and 90° oriented ply lay-ups, for which the off-axis behaviour was experimentally investigated in [33,34,35]. There, the mechanical properties are determined to reach the highest values at 0°, where the fibres are oriented parallel to the loading axis. As the off-axis angle increases, the magnitude of properties decreases until reaching a minimum for an angle of 45°. Afterwards, until reaching an angle of 90°, the properties do increase again slightly.

Finding a behaviour similar to such laminates in discontinuously reinforced composites is very clearly due to the previously described pronounced stratification of fibre orientations in injection moulding, where only the outer portions of the wall thickness are oriented in the longitudinal direction. A significant portion of the core layer however is oriented in transversal direction. 

The values of the material parameters evaluated are listed in Table 1. The shear modulus and shear strength were determined from the results of the diagonal specimens using the Tsai–Hill criterion.

The characteristic values determined here serve as starting values for the cyclic characterisation. 

### 5.2. Results of Cyclic Testing

**Figure 9:**
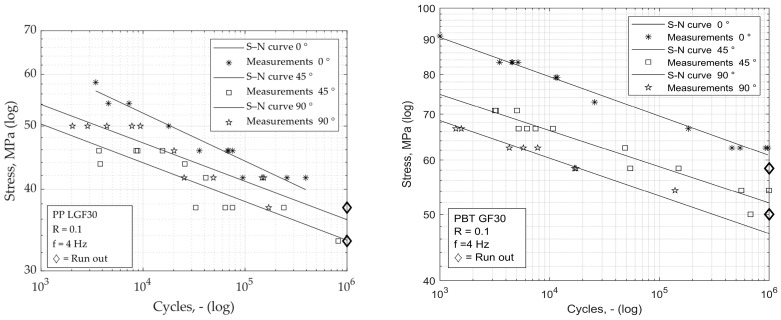
S–N curves for PP LGF30 (**left**) in comparison with PBT GF30 (**right**, [23]), both with 0°, 45° and 90° oriented specimens.

The differently oriented samples were tested using the string-of-pearls method. The left half of Figure 9 shows the S–N curves determined for the respective specimen orientations. As already observed for the static strength, the fatigue strength of the specimens taken transversely from the plates is also higher than that of the diagonally oriented specimens. The slope of the S–N curves differs slightly, but more clearly than was the case with the previously considered short-glass-fibre-reinforced PBT GF30, shown on right side of Figure 9.

**Figure 10:**
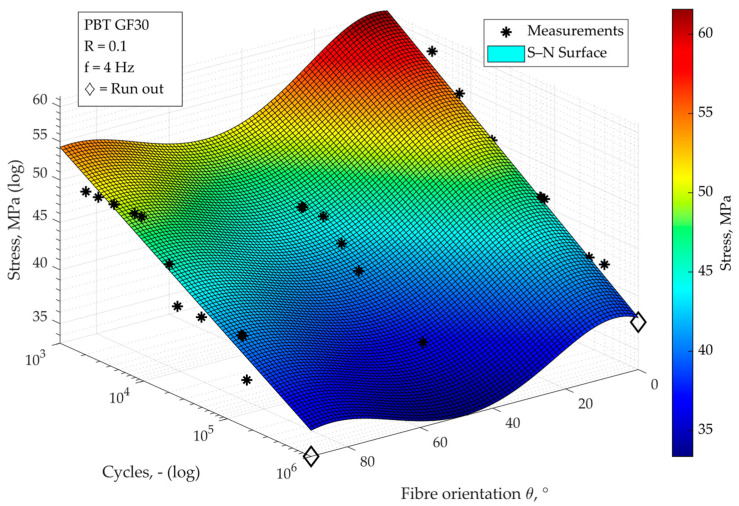
S–N surface interpolation for arbitrary orientation angles in PP LGF30.

Not all arbitrary fibre orientations can be tested, but for the prediction of an arbitrary orientation-dependent fatigue strength, a continuous relationship is necessary, where an S–N surface is formed, which is shown in Figure 10.

From the fact that the fatigue strength is weaker for an orientation of 45° than for 90°, it must be stated that the assumption of a strictly monotonically decreasing behaviour with an increasing orientation angle must be rejected. However, the approach used for modelling, which is based on the Tsai–Hill failure model, allows for this. Instead of a constant falling slope of the surface, a polynomial formulation dependent on the orientation is now chosen:

(4) $$\sigma \left(\theta \right)={\left[\frac{\cos{\left(\theta \right)}^{2}\cdot \left(\cos{\left(\theta \right)}^{2}-sin{\left(\theta \right)}^{2}\right)}{\sigma_{∥,f}^{2}}+\frac{sin{\left(\theta \right)}^{4}}{\sigma_{⊥,f}^{2}}+\frac{\cos{\left(\theta \right)}^{2}\cdot sin{\left(\theta \right)}^{2}}{\tau_{∥⊥,f}^{2}}\right]}^{-\frac{1}{2}}\cdot N^{b(\theta )}$$

where

(5) $$b\left(\theta \right)=p_{1}\theta^{2}+p_{2}\theta +p_{3}$$

The parameters are computed by means of curve-fitting. Their computed values are denoted in Table 2.

The S–N surface calibrated in this way achieves a good prediction quality with a coefficient of determination *R*^2^ of 0.89.

### 5.3. Inducing of Fatigue Damage into Specimens

In order to specifically pre-damage individual specimens, experiments were carried out with a combination of stress and number of cycles that can be assumed not to lead to failure of the specimen. The damage to the specimens is determined by comparing the remaining stiffness after passing through n cycles with the initial stiffness. The determined loss of stiffness is shown in the following Table 3. 

### 5.4. Residual Strength of Pre-Damaged Specimens

**Figure 11:**
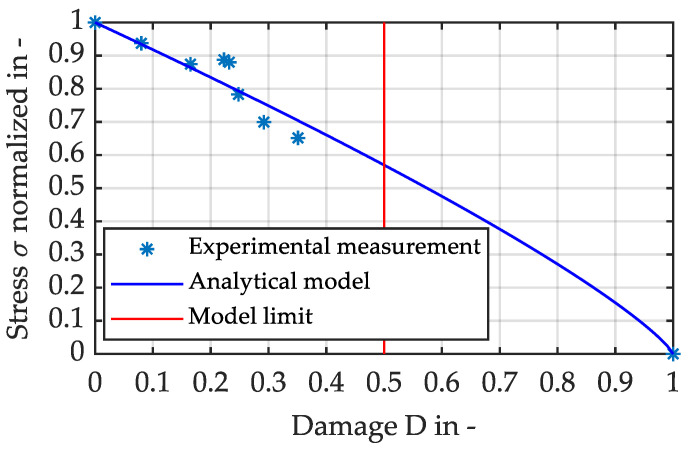
Normalised residual strength over prior fatigue damage in specimen of PP LGF30.

The samples damaged in the previous step were destructively tested here for their residual strengths. These are shown in Figure 11, normalised against the mean initial values of the virgin samples. It can be clearly seen that the residual strength decreases with increasing damage: among the samples examined, there are cases of more than a 30% decrease in residual strength compared to the initial value. 

What is also shown in Figure 11 is the model prediction for the residual strength, which can be calibrated very well for the measured data. It obeys the previously described equation according to Paepegem, Shokrieh and Lessard with an exponent *p* of 0.811:

(6) $$\sigma_{RT}=X_{T}\cdot {\left(1-D\right)}^{p}$$

## 6. Discussion 

**Figure 12:**
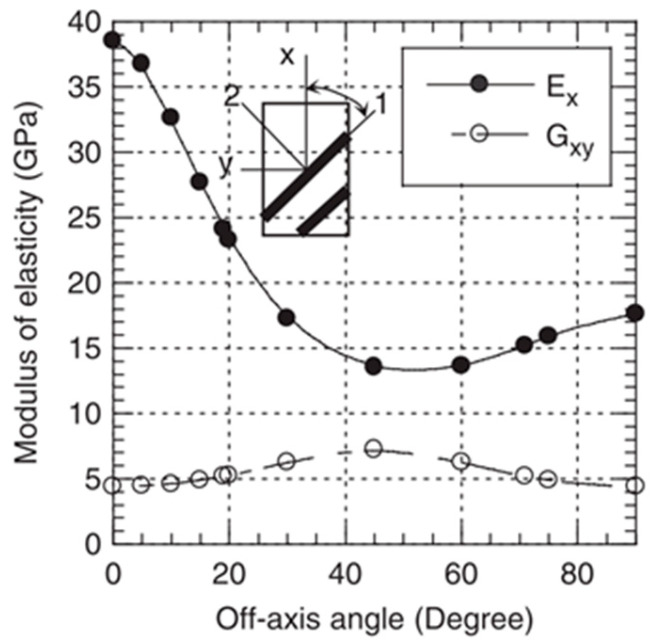
Dependency of mechanical properties on the off-axis angle of 0°/90° for glass-fibre-reinforced laminate [35].

The tests carried out on the long-fibre-reinforced thermoplastic PP LGF30 were able to show that the distinctive, layer-wise orientation of the fibres parallel and transverse to the melt flow has a clear influence on the material behaviour. While in previous studies on the short-fibre-reinforced PBT GF30 the transversely oriented samples performed significantly worse than those taken diagonally, the PP LGF30 showed that the transverse direction provided stronger characteristic values than the diagonal direction. This behaviour can be observed in the statically determined characteristic values for stiffness and strength as well as in the orientation-dependent fatigue strength. It is in accordance with observations for another group of composites, namely ply laminates with layer orientations of 0° and 90°, shown in Figure 12. 

As outlined in Section 3, there is a clear pronouncement of a layered fibre orientation along the longitudinal and transverse directions in the tested material, with the layers that are oriented longitudinally taking up the greater proportion of the cross-section. This results in the effect seen here in which specimens tested in the longitudinal orientation show the best mechanical performance, followed by the transverse orientation. In the case of the 45°-oriented specimens, a reduced reinforcing effect is present in all layers, which then cannot be absorbed by other layers either. It is therefore not surprising that this orientation shows the lowest mechanical performance, both in the static and cyclic range.

The approach of continuous modelling of fatigue behaviour, which could be derived and validated for short-fibre-reinforced thermoplastics, was now intended to predict the continuous behaviour of fatigue strength with varying fibre orientation also for the long-fibre-reinforced thermoplastic. Since the 45° orientation shows the weakest behaviour with this material, there is no strictly monotonously falling relation between 0° and 90° here. This behaviour can nevertheless be successfully predicted by the identified approach, which is based on the Tsai–Hill failure criterion. With regard to the slope of the surface in the N-direction, the approach in its initial form reaches its limits: because of the different slopes at different orientations, the assumption of a constant, average slope for the entire surface cannot be supported here. With an adaptation of the model equation, which now allows for a variable slope dependent on the specimen orientation, a much more accurate modelling of the fatigue behaviour can be achieved.

Finally, samples were specifically pre-damaged by non-destructive cyclic experiments and their residual strength was investigated in subsequent destructive tensile tests. It can be observed that the effects of material deterioration observed with short-fibre-reinforced thermoplastics also occur with the long-fibre-reinforced PP LGF30. The model that was developed to predict the residual strength depending on the damage could also be successfully used for the long-fibre-reinforced material.

## 7. Summary and Conclusions

The influence of fatigue on the residual strength of short- and long-glass-fibre-reinforced injection moulded thermoplastics has been researched little so far. The authors have already presented an approach for modelling the material behaviour after fatigue damage due to service load cases. The aim of this approach is to be able to describe the failure behaviour as a function of the damage. The aim is to show whether the approach developed for short-fibre-reinforced thermoplastics can also be transferred to neighbouring material classes. 

In this paper, experimental investigations were therefore carried out to describe the static and cyclic material behaviour. The remaining residual strength was investigated on specially pre-damaged samples. The methods of the approach according to Witzgall and Wartzack were applied to the determined data and provided very useful results: both the continuous modelling of the fatigue behaviour with Witzgall’s S–N Surface method and the prediction of the residual strengths could be successfully applied.

It can thus be stated that the observations made on the material class of short-fibre-reinforced thermoplastics with regard to fatigue damage and its influence on the residual strength also apply to long-fibre-reinforced thermoplastics. The modelling approach developed for the short-fibre-reinforced thermoplastics could be used successfully, partly unchanged and partly after generalisation, for the prediction of the material behaviour of the long-fibre-reinforced thermoplastics. 

Thus, it seems obvious that the method could be generalised for discontinuously fibre-reinforced injection-moulded thermoplastics, regardless of their fibre length. This will be checked and validated in future studies with further materials in order to achieve a good substantiation for the approach.

## Figures and Tables

**Table 1:** Material parameters determined for PP LGF30 from static testing.

$E_{∥}$	$E_{⊥}$	$\nu_{∥⊥}$	$G_{∥⊥}$
MPa	MPa	-	MPa
5124	5352	0.31	1587

**Table 2:** Fatigue life parameters of the S–N surface.

Parameter	Value	95% Confidence Interval
$\sigma_{∥,f}$, MPa	99.2	83.6–114.9
$\sigma_{⊥,f}$, MPa	82.7	70.3–95.1
$\tau_{∥⊥,f}$*,* MPa	43.0	31.8–54.1
$p_{1}$	−2.8 × 10^−6^	−1.4 × 10^−5^–8.3 × 10^−6^
$p_{2}$	3.5 × 10^−4^	−6.8 × 10^−4^–1.4 × 10^−3^
$p_{3}$	−7.0 × 10^−2^	−8.5 × 10^−2^–−5.5 × 10^−2^

**Table 3:** Material parameters determined for PP LGF30 during damage initiation.

Orientation, °	Stress, MPa	Cycles	Damage
90	50	1500	0.165
90	50	2500	0.351
45	45.8	2500	0.223
45	45.8	3500	0.232
45	41.7	3400	0.080
0	41.7	100,000	0.248
0	41.7	150,000	0.292

## Data Availability

Not applicable.
